# Effects of Rubber Size on the Cracking Resistance of Rubberized Mortars

**DOI:** 10.3390/ma12193132

**Published:** 2019-09-25

**Authors:** Yong Yu, Han Zhu

**Affiliations:** 1 School of Civil Engineering, Qingdao University of Technology, Qingdao 266033, China; 2 Key Laboratory of Coast Civil Structure Safety, Tianjin University, Ministry of Education, Tianjin 300350, China

**Keywords:** rubberized cement-based mortar, rubber particle size, strength, restrained ring shrinkage test, restrained squared eccentric ring shrinkage test

## Abstract

This study investigated the cracking resistance of rubberized cement-based mortars. Three rubber particle sizes were used: Rubber A (major particle size 2–4 mm), Rubber B (major particle size 1–3 mm), and Rubber C (major particle size 0–2 mm). Traditional restrained ring shrinkage test (RRST), new restrained squared eccentric ring shrinkage test (RSERST), mechanical test, and scanning electron microscopy test were conducted. Results showed that the cracking inhibitory effect of Rubber B was the highest among the three rubber particle sizes. SEM results revealed that the particle size of the rubber does not have much effect on the ITZ (interfacial transition zone) position of rubber and cement paste. For the strength differences of the three types of rubberized mortar, it is mainly because the specific surface area increased as the rubber size decreased, which lead to more ITZ positions and pore structures. Our study verified that RSERST can predict the cracking position and shorten the test period. Compared with RRST, RSERST can also increase the restriction degree. $K_{R}$ is defined as the intensification factor of RSERST restriction degree. The average intensification factor is $\bar{K_{R}}=1.17$.

## 1. Introduction

Cracking under restrained shrinkage is a common cause of distress in concrete walls, slabs, and pavements [1]. Cracks in turn lead to leaks, corrosion of rebars, freeze-thaw damage, so as other durability issues [2,3,4], while cement-based materials are brittle and sensitive to shrinkage cracking. For cement-based composites, when the shrinkage of the material is restrained by steel bars or form work, tensile stress develops in materials. The development of tensile stress can then result in an early age cracking, if the tensile stress is higher than the tensile strength [5]. A major contribution of incorporating rubber wastes to cement-based materials is the improvement of the flexibility, toughness and fatigue resistance of the material [6,7,8,9], although the compressive strength of the cement-based material is reduced [10,11]. The use of rubber aggregates is also a suitable solution to improve the cracking resistance of cement-based composites [11,12,13,14]. Recent research [15] shows that rubberized cement-based material is a promising pavement material for the excellent performance on cracking resistance of the material. 

However, rubber particles exist in different sizes that affect rubberized cement-based materials to varying degrees. Sukontasukkul [16] investigated the sound and thermal properties of rubberized concrete with two different size of particle. Sukontasukkul and Tiamlom [17] demonstrated that various properties, such as expansion and shrinkage of rubberized concrete depend strongly on the size and particle shapes of crumb rubber. Su [18] examined the fresh density, strengths, and water permeability of rubberized concrete with different sizes of rubber. Mehmet Gesog [19] analyzed the abrasion and freezing–thawing resistance of concrete with rubbers of different sizes. Arin [20] evaluated the rubber size effects on the water absorption of cement mixtures. Feng [21] determined the effects of rubber size on the impact performance of concrete. If we want to promote rubber cement base material as the pavement material, the kind of rubber size should be first determined. However, knowledge regarding the influence of rubber size effects on the cracking resistance of cement-based materials is still limited. Research is carried out based on this issue in this paper.

Restrained ring shrinkage test (RRST) [22,23,24] is widely used to quantify the cracking resistance of cement-based materials. Its test apparatus is simple, and specimens are circular and axially symmetric. Therefore, its theoretical computation is simple. However, this type of symmetry also produces an uncertain cracking point because cracks due to shrinkage could appear at random in any point within 360° along the ring circumference. A new restrained squared eccentric ring shrinkage test (RSERST) is introduced to improve RRST [25]. In RSERST, stress is concentrated at a certain cracking point and the test cycle is reduced. A test cycle is mostly reduced because of an increase in restriction degree. Nevertheless, the restriction degree of RSERST has been disregarded in a previous study [25].

The present study aims to enhance our understanding on the shrinkage cracking properties of cement rubber mortars derived from rubber of various sizes. RRST and RSERST were performed, and the restriction degree of RSERST was investigated.

## 2. Experiments

### 2.1. Materials and Mix Proportions

**Figure 1:**
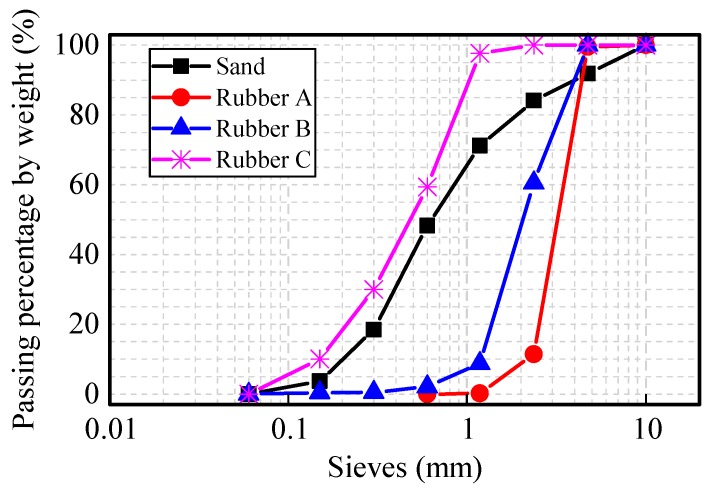
Particle size distribution of fine aggregates.

The cement employed in this test was ordinary Portland cement of Chinese grade 42.5 and was camel brand cement produced in Tianjin (China). Physical properties of used materials are listed in Table 1. The chemical composition of the cement is listed in Table 2. The sand used was local river sand. Tap water was utilized. All of the rubber aggregates were obtained from mechanically ground waste tires. The chemical composition of the crumb rubber is listed in Table 3. Three types of rubber particles were used in this work and were designated as Rubbers A, B, and C. The analyses of the aggregates’ sizes were carried out using the sieve method. The particle size distributions of the fine aggregates are provided in Figure 1. The mix proportions of the rubberized mortars are presented in Table 4.

### 2.2. RSERST

**Figure 2:**
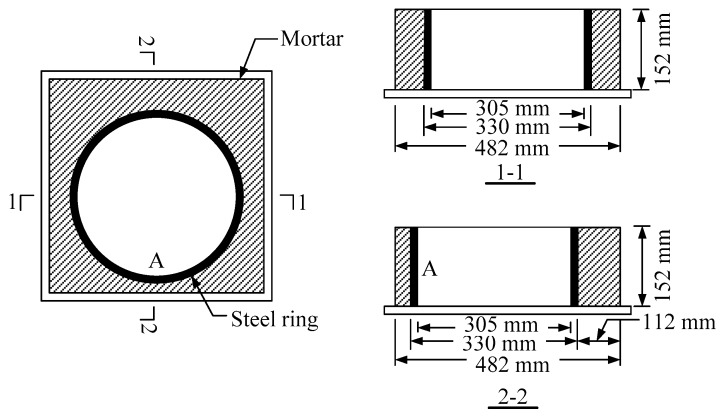
Test device of restrained squared eccentric ring shrinkage test (RSERST).

**Figure 3:**
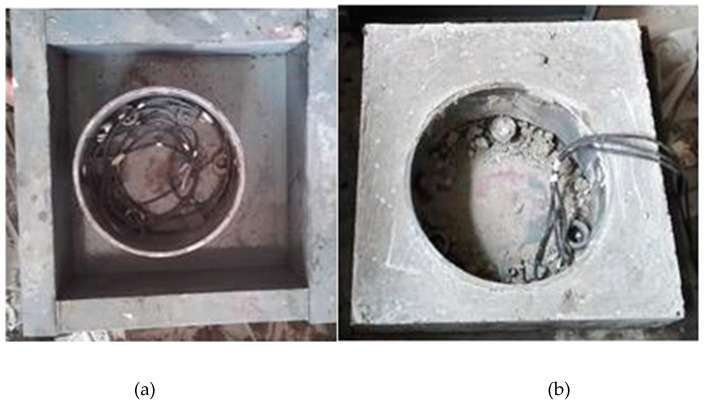
Test specimen of RSERST. (**a**) RSERST molds; (**b**) RSERST specimen.

Dimensions and specimens of RSERST are shown in Figure 2 and Figure 3. Mortar specimens were stripped from the steel molds after 1 day, and the RSERST specimen was covered on the upper surface by waterproof silicone rubber. The specimens were then naturally cured. The temperature of curing room is 20 ± 2 °C, and relative humidity is 50% ± 5%. The strain values were measured every 15 min by three strain gauges pasted at point A, as shown in Figure 2. A total of 21 RSERST specimens were tested.

### 2.3. RRST

**Figure 4:**
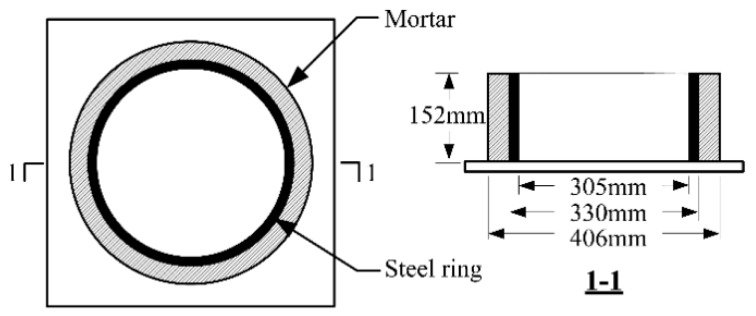
Test device of restrained ring shrinkage test (RRST).

**Figure 5:**
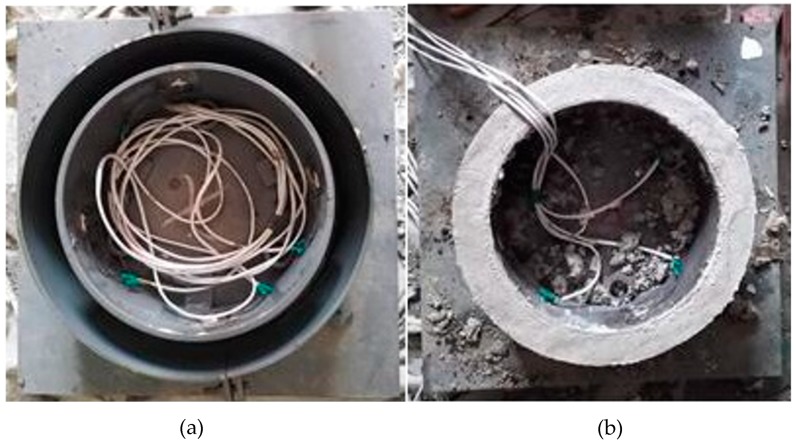
Test specimen of RRST. (**a**) RRST molds; (**b**) RRST specimen

To quantify the cracking resistance of each mortar mixture and to compare the RSERST results, we applied the RRST in accordance with ASTM C1581-04, as shown in Figure 4 and Figure 5. The strain values were measured every 15 min using four strain gauges bonded at the middle location of the inner surface of the steel ring. A total of 21 specimens were casted, and average strain values were calculated.

**Figure 6:**
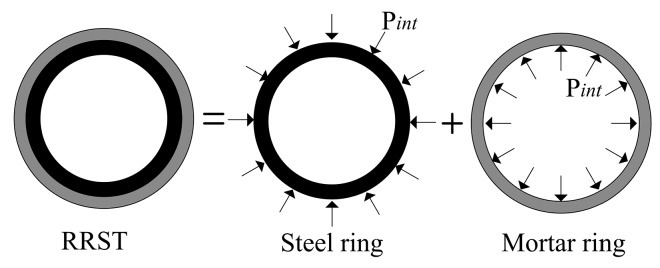
Illustration for computing stress.

The development of strain within the inner surface of the steel ring can be transformed to the maximum circumferential tensile stress of mortar, which occurs at the interface of the mortar and steel through the calculation diagram, as shown in Figure 6 and the following equations:

(1) $$P_{\mathrm{int}}\left(t\right)=-\varepsilon_{ST}\left(t\right)\times E_{Steel}\frac{R_{OS}^{2}-R_{IS}^{2}}{2R_{OS}^{2}}$$

(2) $$\sigma \left(r\right)=P_{int}\frac{R_{IM}^{2}}{R_{OM}^{2}-R_{IM}^{2}}\left[1+\frac{R_{OM}^{2}}{r^{2}}\right]$$

(3) $$\sigma_{max}=-\varepsilon_{ST}\left(t\right)E_{Steel}\left(\frac{R_{IM}^{2}+R_{OM}^{2}}{R_{OM}^{2}-R_{IM}^{2}}\right)\left(\frac{R_{IM}^{2}-R_{IS}^{2}}{2R_{IM}^{2}}\right)$$

where $P_{int}$ is the fictitious interface pressure, $\sigma_{r}$ is the tensile stress in the mortar ring at any point along the radius, $R_{IM}$ is the inner diameter of the mortar ring, is the outer diameter of the mortar ring, $R_{IS}$ is the inner diameter of the steel ring, $R_{OS}$ is the outer diameter of the steel ring, $\varepsilon_{ST}$ is the strain in the steel ring, and $E_{Steel}$ is the modulus of elasticity of the steel.

### 2.4. Mechanical Test

Mortar specimens with 70.7 × 70.7 × 70.7 mm dimension were casted for compressive strength testing in accordance with JGJ/T70-2009 [26]. For the compressive strength test, a total of 84 specimens were prepared. The compressive strengths of the specimens were measured on day 28. Statistical analysis was conducted on the basis of compressive strength for quality test. For flexural strength testing, specimens with dimensions of 40 × 40 × 160 mm were casted according to GB/T 17671-1999 [27], and the specimens were examined on days 1, 3, 7, and 28. A total of 84 specimens were casted for flexural strength test. The specimens were cured at 20 °C ± 2 °C and 40% ± 5% relative humidity, and these conditions were the same as those in RRST and RSERST.

### 2.5. SEM Test

The interfacial transition zones (ITZs) of rubber samples and cement paste were observed under a field emission SEM 1530VP at Institute of oceanology, Chinese academy of sciences, Qingdao China. The mortar samples of 10 × 10 × 10 mm dimensions were polished, cleaned, coated with gold, and evacuated prior to observation.

## 3. Results and Discussions

### 3.1. RSERST Results

**Figure 7:**
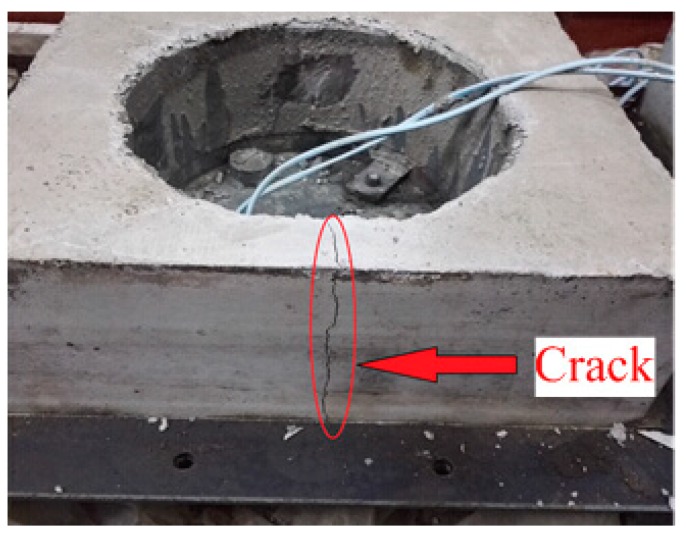
Cracking location of RSERST specimen.

**Figure 8:**
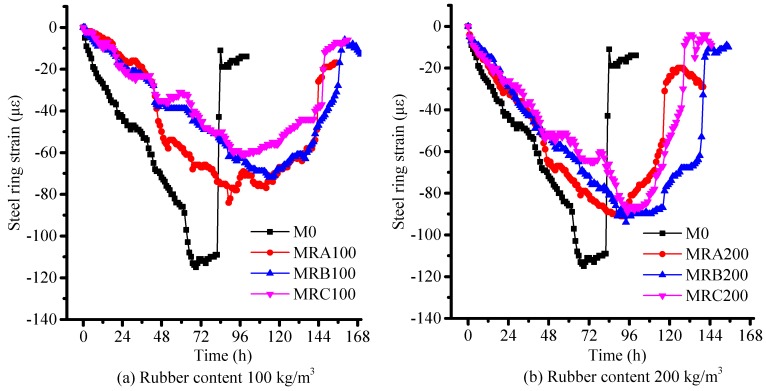
Strain obtained from the RSERST. (**a**) Rubber content 100 kg/m^3^; (**b**) Rubber content 200 kg/m^3^.

The cracks occurred at the thinnest portion of the RSERST specimen, as shown in Figure 7, which verified that RSERST could predict the cracking position. With the predicted cracking position, the observation of the cracks was more convenient. The strains obtained from the steel ring of RSERST are shown in Figure 8. The release of strain on the curves marks the cracking time of the mortar. In terms of size, the effects of Rubber B in preventing cracking are better than that of Rubber A and Rubber C. As for rubber content, the time of cracking increases as rubber content increases.

### 3.2. RRST Results

**Figure 9:**
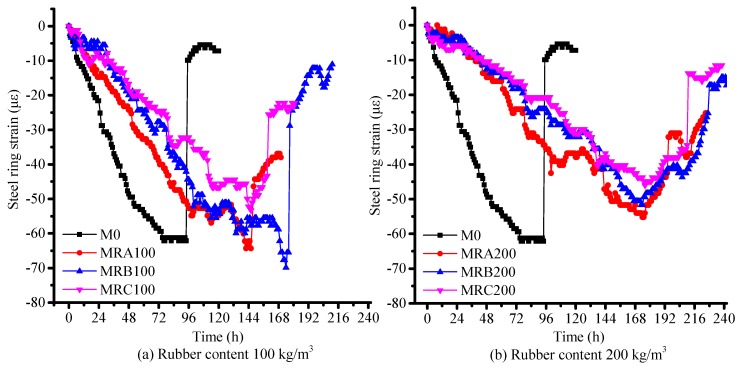
Strain obtained from the RRST. (**a**) Rubber content 100 kg/m^3^; (**b**) Rubber content 200 kg/m^3^.

Figure 9 shows the plots of the strain obtained from the steel ring by RRST as a function of time attained using a data acquisition system. The release of strain on the curves marks the cracking time of the mortar [28,29]. The results clearly demonstrate that the rubber aggregates benefitted from the delayed restrained shrinkage cracking, a finding similar to that of a previous study [12]. With the same rubber contents, the cracking inhibition effect of Rubber B was the highest among those of the three rubber samples tested. The results of RRST are consistent with those of RSERST.

### 3.3. Mechanical Strengths

Table 5 shows the compressive strengths of the seven mixed mortars. The compressive strength decreases as rubber size decreases or rubber content increases. The coefficient value of the compressive strength variation is often used as a control of quality. Day [30] suggested that generally, for a reasonable quality control, a coefficient of variation should between 5% and 10%. Swamy [31] proposed that the limit for fine quality control is 15%. The largest coefficient of variation for the seven mixes is 10.9 for MRC200, which is slightly larger than 10% but much lower than 15%. Therefore, the mortars can be regarded to have good quality.

Table 6 shows the development of flexural strengths of the mixed mortars. In previous studies [4,26], both properties decreased when crumb rubber was incorporated into the mixes. The decline in compressive and flexural strengths was significant when the crumb rubber with small particle sizes was used. The strength reductions may be attributed to the combination of two effects: (1) Lower stiffness in the rubber than in the sand and (2) an increase in the amount of ITZs generated in the mortar with an increase in rubber content or reduction in rubber size. The specific surface area increased as the rubber size decreased and eventually resulted in the reduction in strength when the small crumb rubber particles were utilized.

### 3.4. SEM Results for the ITZ

**Figure 10:**
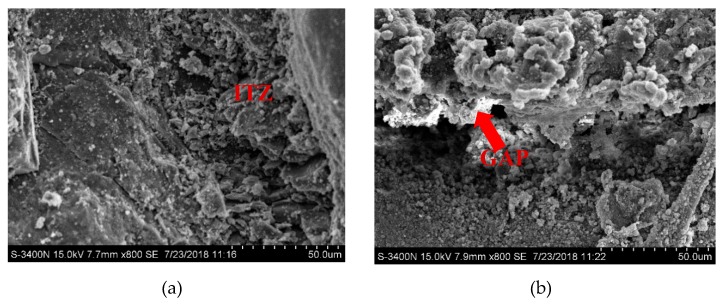

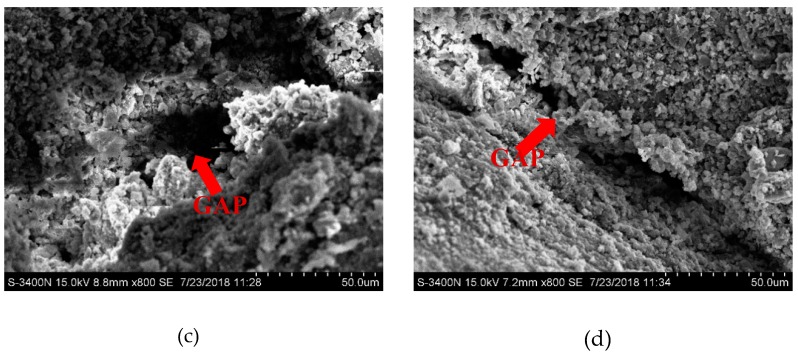
SEM images of rubberized mortar interfacial transition zones (ITZ). (**a**) ITZ of the sand and cement paste; (**b**) ITZ of Rubber A and cement paste; (**c**) ITZ of Rubber B and cement paste; (**d**) ITZ of Rubber C and cement paste.

The SEM images of the fine aggregates and cement matrix interfaces are shown in Figure 10. Only in the ITZ of the sand and cement paste, as shown in Figure a, was the main gap not obvious. In the ITZ of Rubber A, B, C and cement paste, minor cracks grew wider and enlarged to a main gap with large number of pore structures. The it can be concluded that the bonding interface between sand and cement is better than that between rubber and cement. The addition of rubber could reduce the strength of mortar mainly in two aspects: (1) Rubber particles are softer than sand, (2) Rubber particles could introduce pores into the mortar, whereas leading to the reduction of the cement-based materials strength.

From Figure 10b–d, it can be concluded that the particle size of the rubber does not affect the thickness of ITZ position very much, for rubber particles of different particle sizes are all made by mechanical grinding, and the production process is the same. If the rubber is of equal volume, the surface area of small size rubber particles is larger than that of rubber with large particle size. So, it can be concluded that, for the strength differences of the three types of rubberized mortar, it is mainly because the specific surface area increased as the size of rubber decreased, which lead to more ITZ positions and pore structures. So, the basic mechanical properties of MRC200 group with the smallest rubber particles and the largest rubber content are the worst.

### 3.5. Discussions

#### 3.5.1. Comparison of RSERST and RRST

The cracking time of RRST and RSERST specimens are shown in Table 7. Results of RSERST are in agreement with those of RRST. The order of cracking in RSERST consistently matches with that in RRST. Both results show that 0.5M0 cracked first, followed by the cracking of 0.5MRA100, 0.5MRC100, 0.5MRB100, 0.5MRA200, 0.5MRC200; 0.5MRB200 cracked the last. Compared with RRST, RSERST can shorten the test period, and the longer period of cracking results in more time that is saved by REERST. Figure 8 and Figure 9 also demonstrate that the strains of the seven mixes obtained from RSERST were larger than those obtained from RRST, which possibly resulted from the stress concentration of RSERST.

#### 3.5.2. Comparison of Restraint Circumferential Stress and Flexural Tensile Strength

**Figure 11:**
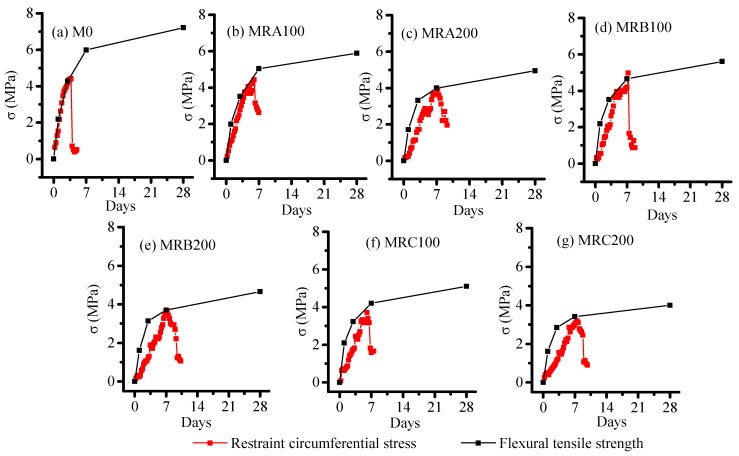
Comparison of restraint circumferential stress and flexural tensile strength.

Figure 11 shows the development comparison of restraint circumferential stress and flexural tensile strength. The mortar cracks when the corresponding hoop constraint stress is greater than the tensile strength. Rubber can delay mortar cracking because the strength development speed of mortars containing rubber is quicker than the hoop constraint stress development speed of the same sample. With the same rubber contents, the cracking inhibition function of Rubber B was better than those of Rubbers A and C.

#### 3.5.3. Intensification Factor of RSERST Restriction Degree $K_{R}$

We define $K_{R}$ as the intensification factor of RSERST restriction degree, which shows the maximum constraint stress ratio of RSERST and RRST with the same materials and test conditions, as shown in Equation (4).

(4) $$K_{R}=\frac{\sigma_{RSERST}}{\sigma_{RRST}}$$

where $\sigma_{RSERST}$ is the maximum constraint stress of RSERST specimen, and $\sigma_{RRST}$ is the maximum constraint stress of RRST specimen.

When the RSERST specimen cracks, the $\sigma_{RSERST}$ equals the tensile strength of the mortar. For the $\sigma_{RRST}$ taken at the same time, the variable can be calculated through Equation (3), as shown in Table 8. Then, $K_{R}$ can be calculated by using Equation (4). $K_{R}$ of the mortars at the cracking time of the RSERST specimen are shown in Table 8. We can conclude that $K_{R}$ of the mortars is >1, and the average $K_{R}$ is 1.17, which indicates that the eccentricity of the RSERST increases the restriction degree of the mortars compared with that of RRST. 

## 4. Conclusions

The cracking properties of rubberized mortars with rubber of different sizes and contents were systematically investigated, and the restriction degree of RSERST was calculated. Based on our results, the conclusions can be drawn as follows:

1. RSERST can predict the cracking position and shorten the test period, and the restriction degree is higher in RSERST than in RRST. The average intensification factor is $\bar{K_{R}}=1.17$.
2. Both RRST and RSERST revealed that the addition of rubber can delay cracking. The content and size of rubber can both contribute to the cracking resistance of rubberized mortars. With rubber of equal content, the cracking inhibitory effect of Rubber B is higher than that of Rubbers A and C.
3. The bonding interface between sand and cement is better than that between rubber and cement. The particle size of the rubber does not affect much on the ITZ position of rubber and cement paste. For the strength differences of the three types of rubberized mortar, it is mainly because the specific surface area increased as the rubber size decreased, which lead to more ITZ positions and pore structures.
4. The addition of rubber will inhibit the development of mortar tensile strength. With rubber particles of a smaller size, more additional pores are introduced, leading to more obvious reduction effects. While, as the rubber is a soft filling, with a smaller particle size, the rubber distribution is more uniform, leading to better cracking inhibition effect. The effect of rubber particle size is opposite in two aspects. Therefore, rubber particle B, which is of medium size, performed best in the cracking inhibition.

## Figures and Tables

**Table 1:** Physical properties of used materials.

Properties	Cement	Sand	Rubber
Specific density (kg/m^3^)	3100	2650	1050
Water absorption (%)	–	1.3	0.35

**Table 2:** Chemical composition of P.O42.5 grade ordinary Portland cement.

Chemical Compound	CaO	SiO_2_	Al_2_O_3_	Fe_2_O_3_	SO_3_	MgO	Lgnition Loss
Percentage (%)	63.11	22.60	5.03	4.38	2.24	1.46	1.18

**Table 3:** Chemical ingredients of crumb rubber (mass fraction %).

Rubber Hydrocarbon	Carbon Black	Acetone Extract	Isoprene	Water	Ash Content	Fiber Content	Metal Content	Others
45.2	25.8	14.2	12.1	0.8	0.9	0.5	0.08	0.42

**Table 4:** Mix proportions of mortars (values in mass proportion).

Mix	Rubber	Cement	Water	Sand
M0	0	2	1	5
MRA100	0.33	2	1	4.17
MRA200	0.67	2	1	3.33
MRB100	0.33	2	1	4.17
MRB200	0.67	2	1	3.33
MRC100	0.33	2	1	4.17
MRC200	0.67	2	1	3.33

**Table 5:** Compressive strength of mortars (MPa).

NO.	M0	MRA100	MRA200	MRB100	MRB200	MRC100	MRC200
1	43.5	30.5	17.5	30.0	16.5	22.7	12.1
2	43.3	29.7	19.6	26.4	17.7	20.1	12.0
3	43.2	29.3	19.5	28.9	17.1	21.4	13.5
4	42.9	28.8	19.0	29.4	17.1	20.4	12.7
5	42.7	28.5	20.3	29.5	16.3	19.1	13.2
6	42.3	28.5	18.2	27.0	16.9	21.8	12.9
7	41.3	28.1	21.0	30.3	19.0	20.5	10.7
8	42.7	27.6	17.7	26.5	20.8	20.8	11.3
9	41.0	27.3	20.7	24.2	17.2	19.6	11.0
10	40.5	26.9	22.7	29.5	16.5	19.9	13.7
11	39.8	26.1	19.8	27.3	18.4	17.8	11.9
12	36.1	25.7	15.9	29.7	17.5	18.8	9.2
Mean (*x*)	41.6	28.1	19.3	28.2	17.6	20.2	12.0
SD (σ)	2.09	1.40	1.81	1.90	1.29	1.36	1.31
COV (σ/x) %	5.03	5.12	9.39	6.74	7.31	6.72	10.90

**Table 6:** Flexural strength of mortars (MPa).

NO.	M0	MRA100	MRA200	MRB100	MRB200	MRC100	MRC200
1d	2.2	2.0	1.7	2.2	1.6	2.1	1.6
3d	4.3	3.5	3.3	3.5	3.1	3.2	2.9
7d	6.0	5.0	4.0	4.7	3.7	4.2	3.4
28d	7.2	5.9	4.9	5.6	4.7	5.1	4.0

**Table 7:** Cracking time of RSERST and RRST specimen (h).

	M0	MRA100	MRA200	MRB100	MRB200	MRC100	MRC200
RSERST	82.25	116.25	143.50	139.25	157.00	128.25	147.00
RRST	94.50	147.25	194.50	176.50	227.75	160.00	210.50
Time saved	12.25	31.00	51.00	37.25	70.75	31.75	63.50

**Table 8:** $K_{R}$ of the mortars at the cracking time of the RSERST specimen.

Sample	$\sigma_{RSERST}\;(\mathrm{MPa})$	$\sigma_{RRST}\;(\mathrm{MPa})$	$K_{R}$
M0	4.69	4.36	1.08
MRA100	4.33	4.00	1.08
MRA200	4.21	3.36	1.25
MRB100	4.47	4.03	1.11
MRB200	3.76	3.30	1.14
MRC100	3.92	3.16	1.24
MRC200	3.50	2.74	1.28
Mean ($\bar{K_{R}}$)			1.17
SD (σ)			0.09
COV (σ/$\bar{K_{R}}$)			0.07
